# Reflections of the 1944 Ahiska exile on Ahlat cuisine

**DOI:** 10.3389/fnut.2026.1903820

**Published:** 2026-08-14

**Authors:** Neslihan Serçeoğlu, Erkan Denk, Yener Oğan, Umut Güleryüz

**Affiliations:** 1 Department of Gastronomy and Culinary Arts, Faculty of Tourism, Atatürk University, Erzurum, Türkiye; 2 Department of Tourism Management, Kanik School of Applied Sciences, Bitlis Eren University, Bitlis, Türkiye; 3 Department of Gastronomy and Culinary Arts, Kaman School of Applied Sciences, Kirsehir Ahi Evran University, Kirsehir, Türkiye

**Keywords:** 1944 Ahiska exile, Ahlat, cultural memory, heritage, migration

## Abstract

**Introduction:**

Following the forced deportation of 1944 under Soviet rule, Ahıska Turks were dispersed across various regions, seeking to preserve their cultural identity despite repeated displacement. This study examines the reflections and continuity of Ahıska culinary culture within Ahlat (Bitlis) cuisine. Specifically, it investigates how traditional culinary practices have been preserved, transformed, and adapted through interactions with the local culinary culture of Ahlat, exploring how this community preserved its identity through foodways after exile.

**Methods:**

Adopting a qualitative, descriptive, and exploratory research design, data were collected through semi-structured face-to-face interviews. Using purposive sampling, 18 participants of Ahıska ethnic origin residing in Ahlat were interviewed. The collected data were transcribed verbatim and analyzed using qualitative content analysis across five predefined core themes: Migration, Food and Beverage, Culinary Practice, Events, and Tradition.

**Results:**

Findings reveal that Ahıska cuisine has largely preserved its traditional characteristics, with staple features centered around pastries, dough-based dishes (such as Hinkal, Kete, and Samsa), and dairy products. Steaming emerged as a distinctive cooking method, and Hinkal was identified as a paramount symbol of culinary identity and cultural continuity. The study found that Ahıska culinary traditions have adapted to and coexisted with local Ahlat food culture without replacing it. Additionally, meals serve as an essential medium for social solidarity and are deeply integrated into life-cycle rituals (e.g., weddings, funerals).

**Discussion:**

Ahıska cuisine functions as a “portable homeland,” enabling displaced individuals to maintain collective memory, cultural identity, and social cohesion across generations. Traumatic migration memories are continually re-encoded into everyday culinary practices. Rather than static retention or cultural erasure, the interplay between Ahıska and Ahlat cuisines demonstrates a dynamic process of culinary adaptation and hybridization, underscoring the vital role of intangible food heritage in diaspora contexts.

## 1 Introduction

Migration, a fate inherent in every geography, can occur either voluntarily or involuntarily (1). Events causing migration include war, exile, economic reasons, disasters, and political, cultural, and social movements. In its most general definition, it is the act of individuals or societies leaving their current geographical location and moving to a new one. Forced migrations inherently involve diversity, and it is important to understand this situation well (2). Forced migration also transforms daily cultural practices, especially food traditions, which play a significant role in preserving identity, memory, and a sense of belonging. For displaced communities, cuisine is not only a means of nourishment but also a form of cultural expression where collective experiences are remembered and passed down through generations. Therefore, recent research examines food within the context of culinary heritage, diaspora, and intangible cultural heritage (3, 4).

As is known, a society’s history, traditions, culture, and dietary habits can be preserved and passed on to future generations through its cuisine. Oğan and Uzman (5) states in his study that culinary culture reflects a society’s traditions, beliefs, identity, heritage, and historical roots. Therefore, culinary culture is dynamic, encompasses a wide historical range, and best represents the culture of societies. Rather than being a static collection of recipes, culinary heritage evolves through migration, adaptation, and interaction with new cultural environments. Consequently, traditional food practices become symbolic expressions of cultural continuity that enable diaspora communities to preserve and reconstruct their identities (6, 7).

One of the most significant forced migration experiences of the twentieth century involving Turkish communities the deportation of the Ahiska Turks on November 14, 1944. As part of the Soviet Union’s wartime deportation policies, Ahiska Turks, together with several other ethnic groups, were forcibly relocated from their homeland to regions such as Uzbekistan, Kazakhstan, and Kyrgyzstan (8). This process resulted in the displacement of approximately 90,000–120,000 people and left lasting demographic, social, and cultural impacts on the community’s collective memory (9). Ahiska, located at a strategic crossroads between the South Caucasus and Eastern Anatolia, has become a symbol of displacement and cultural continuity for the Ahiska Turks. Over time, some members of the community migrated to Türkiye and settled in different regions, including the Ahlat district of Bitlis (10). From this perspective, the experience of the Ahiska Turks provides an important example of how forced migration reshapes culinary practices while simultaneously preserving cultural identity. Despite repeated displacement across different countries, traditional food practices have continued to function as symbolic markers of belonging and collective memory. Accordingly, examining Ahiska cuisine offers an opportunity to understand how culinary heritage is preserved, transformed, and renegotiated within new settlement contexts.

Previous studies indicate that Ahiska remained a Turkish settlement area for centuries and that, despite repeated displacement, Ahiska Turks largely preserved their language, traditions, and cultural identity (8, 11–14). Existing scholarship has predominantly focused on the historical, political, and sociocultural consequences of the Ahiska exile, while culinary culture has generally been addressed only within the broader context of folklore. Studies by Taşdemir (15), Kırzıoğlu (16), and Baddeley (17) highlighted the demographic, socio-psychological, and historical dimensions of exile and displacement, whereas later studies emphasized the continuity of Ahiska cultural identity through family structures, traditions, and everyday practices (18). The existing literature regarding cultural foods specifically concerning the benefits of traditional food security for immigrants and refugees has recently been synthesized (19).

Research directly examining Ahiska food culture remains limited. In a study conducted in Bishkek, Kyzy and Sormaz (20) demonstrated that Ahiska cuisine functions as an important carrier of identity, belonging, and cultural memory through traditional dishes and food-sharing practices. However, their study was limited to the Central Asian context and did not address culinary interactions with local communities in Türkiye. Similarly, Seçim and Kaya (21) examined the adaptation of Ahiska cuisine in different regions of Türkiye and documented the preservation of culinary traditions following migration. Despite the growing literature on the Ahiska exile, limited attention has been paid to cuisine as a mechanism of cultural continuity and identity preservation. Furthermore, existing studies have largely documented traditional foods rather than exploring how culinary practices evolve through interaction with host communities after migration.

Although increasing attention has been paid to the identity and cultural continuity of the Ahiska Turks, studies specifically examining their culinary heritage remain limited. Existing research has largely documented traditional foods and culinary customs within particular geographical contexts, while the interaction between Ahiska cuisine and local food cultures in Türkiye has received comparatively little attention. Consequently, processes of culinary adaptation, continuity, and hybridization remain insufficiently understood. Ahlat provides an appropriate setting to explore these issues, as Ahiska Turks have lived alongside the local population for decades in an environment that shares certain ecological characteristics with their homeland. This context offers an opportunity to examine both the preservation of traditional culinary practices and the mutual influences between Ahiska cuisine and the local food culture of Ahlat. Accordingly, this study addresses the following research questions:

• RQ1. How has the 1944 Ahiska exile influenced the preservation of culinary identity and food-related cultural memory among Ahiska Turks living in Ahlat?

• RQ2. Which traditional foods, culinary practices, rituals, and foodways have been maintained or transformed following settlement in Ahlat?

• RQ3. To what extent have interactions between Ahiska culinary traditions and the local cuisine of Ahlat resulted in processes of adaptation, continuity, and hybridization?

By addressing these questions, the study contributes to the literature on culinary heritage, diaspora foodways, food anthropology, and cultural memory by demonstrating how cuisine functions both as a vehicle for identity preservation and as a dynamic cultural system shaped by migration and local interaction.

## 2 Analysis framework

### 2.1 Ahiska

Ahiska is strategically located in a transition zone at the intersection of the Caucasus, Eastern Anatolia, and the northern Black Sea region. Owing to its fertile agricultural lands, transportation routes, and military significance, the region has historically occupied an important geopolitical position (15, 22). The name Ahiska is associated with the expression “Ak-Sıka” (White Castle) found in the Book of Dede Korkut. Therefore, in some historical studies, Ahiska is also considered a place name of Turkish origin (23). Historical sources indicate that the region has long been inhabited by Turkic communities, while the language, traditions, and social structure of the Ahiska Turks display strong similarities with Anatolian Turkish culture (8, 11, 13, 14).

**FIGURE 1:**
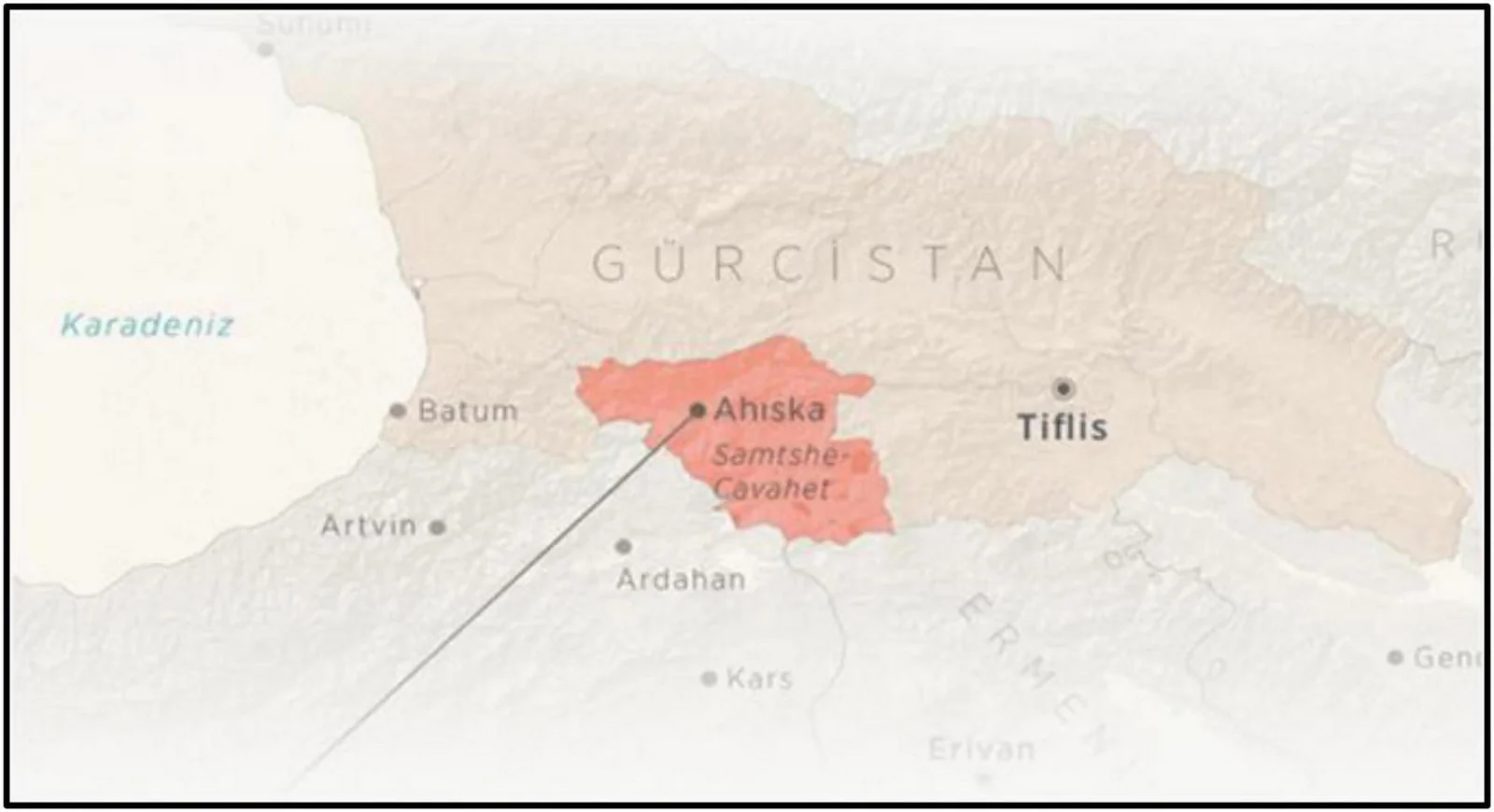
ULUSAM (24). Adapted from (25) with permission from Aegean Geographical Journal.

Historically, Ahiska remained under Ottoman rule until the nineteenth century, when it became part of the Russian Empire following the Russo–Turkish War and the Treaty of Edirne (1829). Subsequent political changes and Soviet nationality policies culminated in the mass deportation of Ahiska Turks on 14 November 1944. This forced exile fundamentally transformed the demographic, cultural, and social structure of the community and resulted in the displacement of thousands of Ahiska Turks to Central Asia (11, 15). Despite repeated displacement and resettlement, Ahiska Turks largely preserved their language, traditions, family structure, and culinary practices. Previous studies have primarily examined the historical, political, and sociocultural dimensions of the exile, whereas culinary culture has generally been addressed only as a component of folklore (18). Consequently, relatively little attention has been paid to understanding cuisine as a mechanism for preserving cultural identity and collective memory following forced migration. From the perspective of food anthropology and culinary heritage, the Ahiska case provides an important opportunity to examine how food practices function as cultural resources that enable displaced communities to maintain social cohesion, reconstruct identity, and negotiate continuity within new settlement environments. Rather than representing only traditional recipes, Ahiska cuisine reflects everyday foodways through which memories of the homeland are preserved and transmitted across generations despite geographical displacement. For this reason, understanding the historical background of Ahiska is essential for interpreting how migration has shaped culinary practices and how these practices continue to function as carriers of cultural identity among Ahiska Turks living in Ahlat. The geographical location of the Ahiska region is presented in Figure 1.

### 2.2 1944 Ahiska exile

During World War II (1939–1945), the Soviet Union subjected several ethnic communities living in border regions to forced deportations. Following a 1944 decision by the State Defense Committee, the Ahiska Turks, together with Crimean Tatars, Volga Germans, and other Caucasian peoples, were deported to Central Asia (8). Approximately 90,000–120,000 Ahiska Turks were forcibly removed from their homeland and transported to Uzbekistan, Kazakhstan, and Kyrgyzstan under extremely harsh conditions (9, 15). Numerous studies report that thousands of people died during the journey because of hunger, cold, disease, and inadequate living conditions (11, 26, 27).

Following the deportation, Ahiska Turks were placed under the “special settlement” system, which imposed severe restrictions on mobility, property ownership, education, and social life (28). The emptied villages were subsequently resettled with other populations, fundamentally transforming the demographic structure of the region (29). Although Soviet authorities justified the deportation on security grounds, many scholars interpret the exile as part of broader demographic and political restructuring policies implemented during the Stalin era (9, 30). Beyond its demographic consequences, the 1944 exile profoundly reshaped the cultural life of the Ahiska community. Dispersed across different countries and later resettled in new regions, Ahiska Turks relied heavily on everyday cultural practices–including language, family traditions, and particularly cuisine–to maintain collective identity. Within this context, food practices became an important means of preserving cultural memory and transmitting intangible cultural heritage despite repeated displacement. Consequently, the Ahiska exile provides a valuable case for examining the relationship between forced migration, cultural continuity, and culinary heritage.

### 2.3 Ahlat and Ahiska cuisine

**FIGURE 2:**
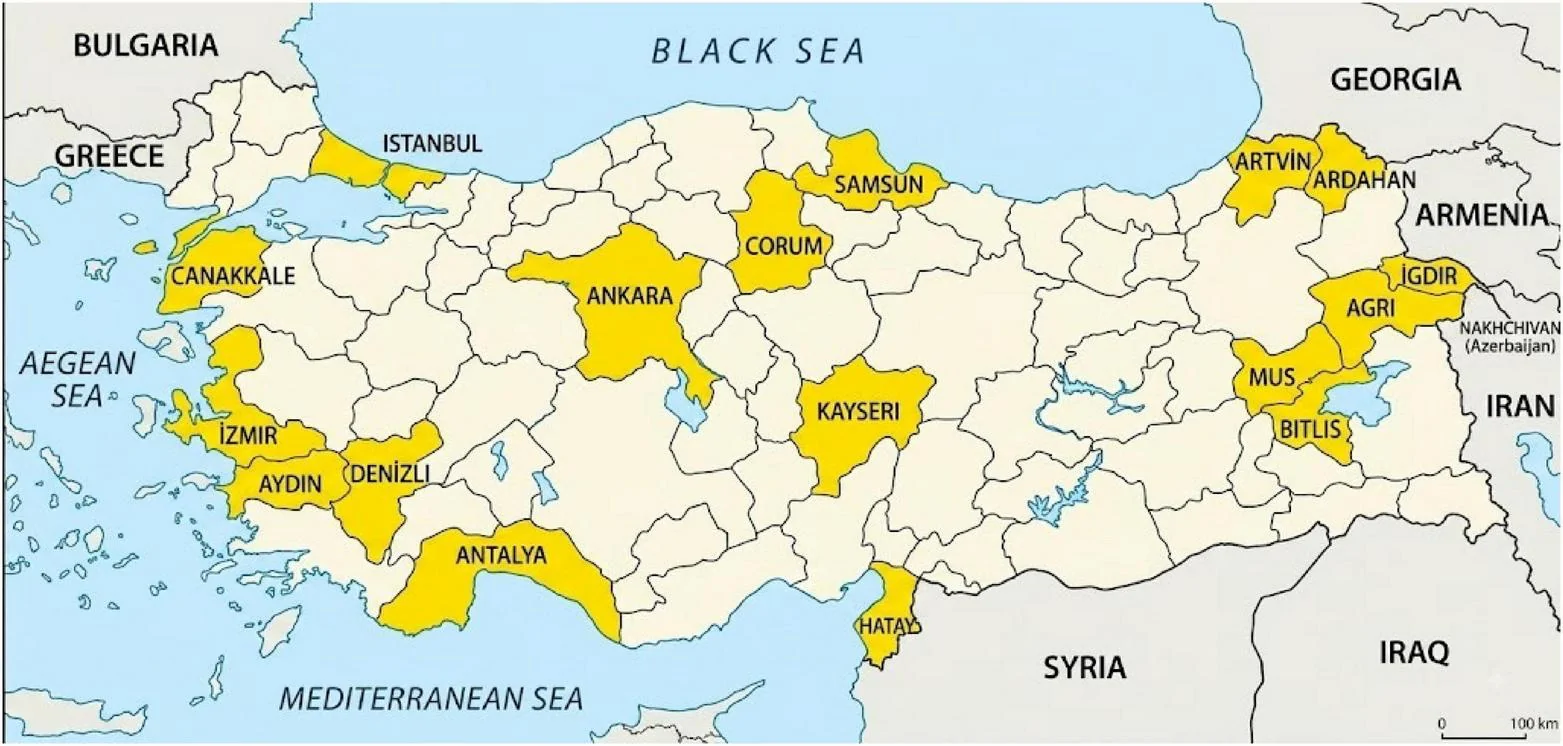
Ahiska Turks of Türkiye. Adapted from (25) with permission from Aegean Geographical Journal.

According to current estimates, approximately 500,000–600,000 Ahiska Turks live in Türkiye (31). Following successive migration waves, many Ahiska families settled permanently in different regions of Türkiye, including the district of Ahlat in Bitlis Province. Since 2016, Ahlat has also become one of the principal settlement areas for Ahiska families evacuated from Ukraine through state-supported resettlement programmes. Consequently, the migration history of the Ahiska Turks represents a multi-stage process extending from the 1944 deportation to Central Asia and subsequently to Türkiye (Figure 2).

Ahlat district, located on the shores of Lake Van, has historically been an important cultural center of Anatolia. In addition to its historical significance, the district possesses a well-established culinary tradition characterized by tandoor cooking, preserved foods, cereal-based dishes, and livestock products (32). These characteristics provide a suitable cultural environment for examining interactions between local culinary traditions and the food heritage brought by Ahiska migrants. Rather than replacing local food traditions, participants’ narratives and the existing literature suggest that Ahiska cuisine has largely coexisted with the local culinary culture of Ahlat. Traditional dishes such as Hinkal, Kete, Haçapur, and various pilafs continue to be prepared within Ahiska households while gradually becoming recognized within the broader local food culture. This process reflects not only the preservation of culinary identity but also gradual adaptation through everyday interaction with the host community. Accordingly, the relationship between Ahiska and Ahlat cuisines should be understood as a process of cultural continuity and culinary adaptation rather than cultural replacement. The coexistence of local and migrant food traditions illustrates how cuisine functions simultaneously as a marker of identity and as a medium through which intercultural interaction and hybridization develop over time. This perspective forms the conceptual basis for analysing the empirical findings presented in this study.

## 3 Methodology

### 3.1 Research design

This study employed a qualitative research design to examine the continuity and transformation of Ahiska culinary culture among Ahiska Turks living in the Ahlat district of Bitlis following the 1944 exile. Specifically, the study investigated how culinary identity has been preserved after forced migration, which traditional food practices have been maintained or transformed, and how interactions between Ahiska and Ahlat cuisines have contributed to processes of cultural continuity, adaptation, and culinary hybridization.

A qualitative approach was considered appropriate because the research aimed to explore participants’ lived experiences, migration memories, cultural meanings, and everyday culinary practices rather than measure predetermined variables. Accordingly, the study was designed within a descriptive and exploratory qualitative framework (33, 34). The research process followed the stages of problem identification, field selection, data collection, analysis, interpretation, and reporting (34, 35).

**FIGURE 3:**
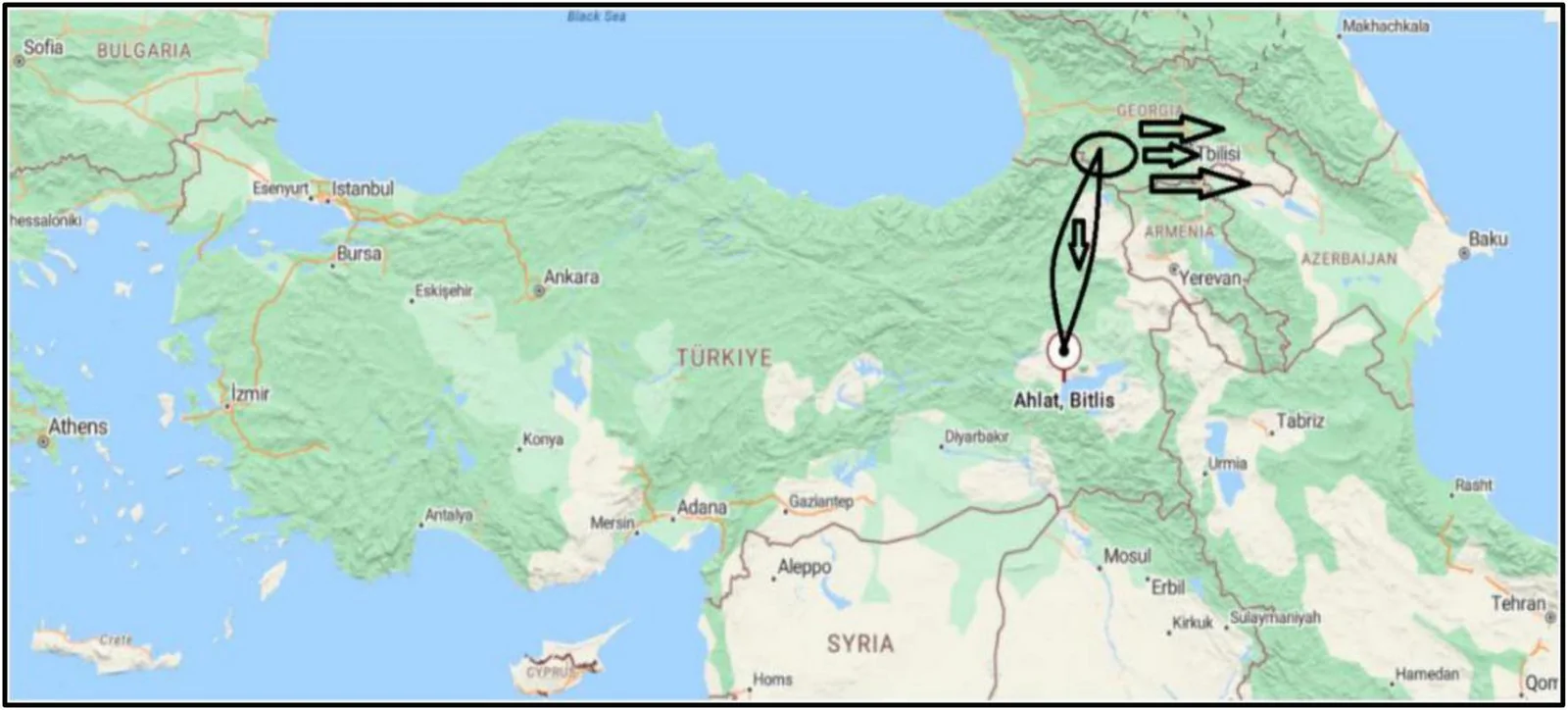
Research field locations.

Ahlat was purposively selected as the research setting because it represents one of the longest-established Ahiska settlement areas in Türkiye and provides an appropriate context for examining both the preservation of traditional Ahiska cuisine and its interaction with local culinary culture. This characteristic directly corresponds to the research questions concerning culinary continuity, adaptation, and cultural interaction following forced migration. The regions associated with the migration process are presented in Figure 3.

### 3.2 Sampling and participants

Purposive sampling was employed to obtain information-rich participants with direct experience and knowledge related to the research phenomenon (36, 37). Participants were recruited through local community networks and recommendations from individuals familiar with the Ahiska community in Ahlat. The inclusion criteria were: (i) being of Ahiska origin, (ii) currently residing in Ahlat, (iii) possessing direct knowledge or lived experience of Ahiska culinary traditions, and (iv) voluntarily agreeing to participate in the study. Particular attention was given to older participants, especially first-generation witnesses of the post-exile period and individuals who had acquired culinary knowledge through intergenerational family transmission.

In total, face-to-face interviews were conducted with eighteen participants between December 1 and December 31, 2025. Interviews were held in locations preferred by the participants to ensure a comfortable environment and facilitate open discussion of migration experiences, food-related memories, and culinary practices. Each interview lasted approximately 40 min.

Data collection continued until thematic saturation was achieved, meaning that additional interviews no longer generated substantially new codes or themes relevant to the research objectives (38). The final sample size of eighteen participants was therefore considered sufficient to provide adequate analytical depth for the study. Prior to data collection, written informed consent was obtained from all participants in accordance with ethical principles. Ethical approval for the study was granted by the Bitlis Eren University Social and Human Sciences Research Ethics Committee (Decision No. 2025/11-13; Approval No. E.8607). Confidentiality and anonymity were maintained throughout all stages of the research process.

### 3.3 Data collection

**FIGURE 4:**
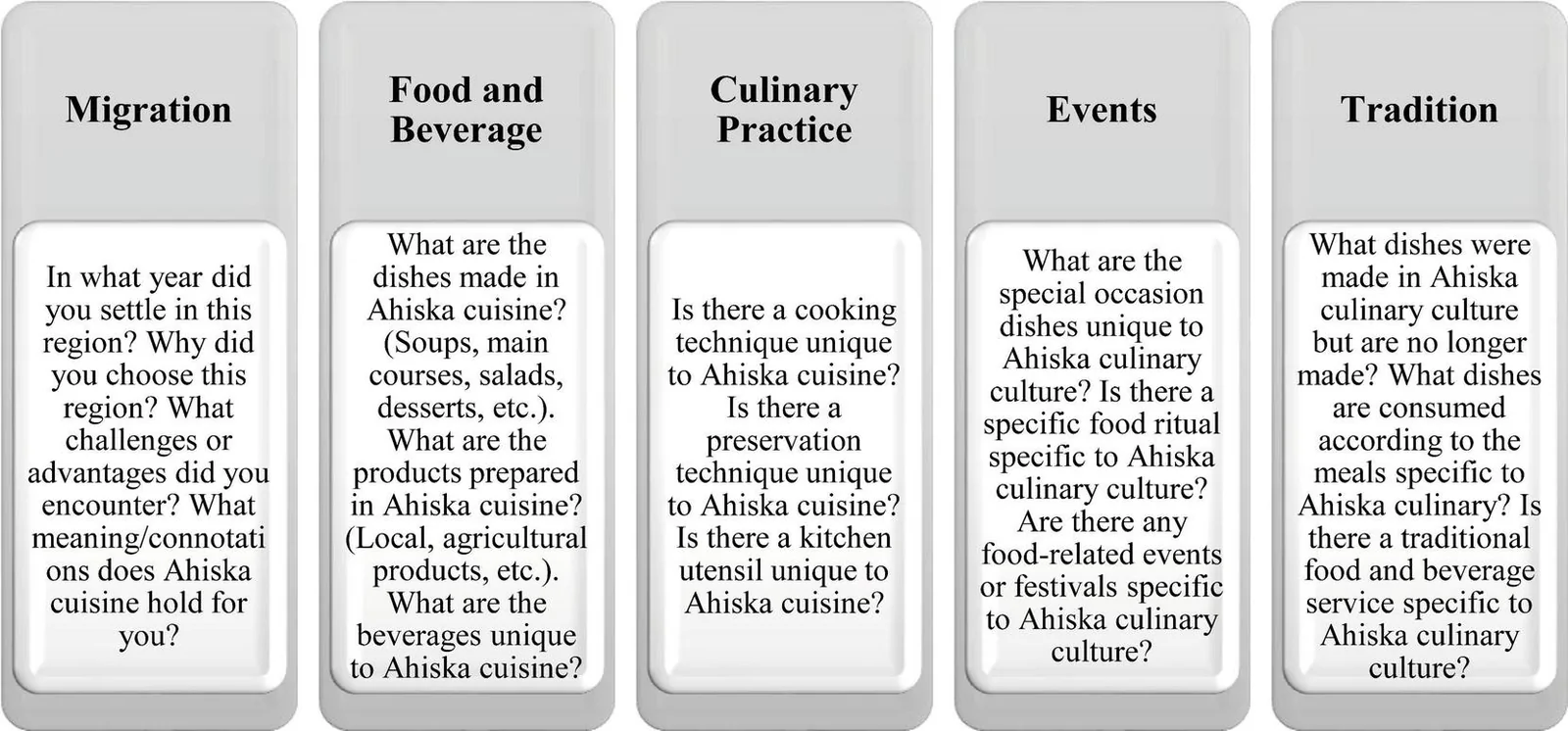
Research themes and questions.

Research data were collected through the semi-structured interview technique, which allows participants to express their memories, experiences, and culinary knowledge in detail. The interview form used in this study was adapted from the research titled Reflections of Türkiye-Greece Population Exchange on Manisa Alaşehir Culinary Culture by Oğan and Uzman (5). The interview protocol was reviewed by two experts in gastronomy and qualitative research to assess the clarity, relevance, and comprehensiveness of the interview questions before fieldwork. Revisions were made based on expert feedback to improve the wording and sequence of several questions. Semi-structured interviews provide flexibility for both the researcher and participants, while also ensuring consistency around the core research themes. The following questions were addressed in the semi-structured interview form, in accordance with the research themes (Figure 4).

As shown in Figure 4, the interview questions were grouped under a total of five main themes: “Migration, Food and Beverage, Culinary Practice, Events, and Traditional.” Through these themes, the study aimed to reveal migration memories, preserved food habits, preparation techniques, food-related rituals, and the transmission of traditional culinary practices across generations. Although the interview guide was organized around these five predefined themes, additional probing questions were asked whenever participants introduced new topics relevant to the research questions. This flexible approach enabled the collection of rich and context-specific narratives while maintaining consistency across interviews. All interviews were audio-recorded with the participants’ consent and transcribed verbatim immediately after data collection. The transcripts constituted the primary dataset used for qualitative analysis.

Following transcription, the interview texts were checked against the original audio recordings to ensure transcription accuracy before the coding process commenced. Personal information about the participants was not disclosed in the study in accordance with ethical principles. Instead, participants were coded as “P1, P2, P3, … P18,” and their demographic information is presented in Table 1.

**TABLE 1:** Demographic characteristics of participants.

*P*	Gender	Age	Marital status	Occupation	Education
P1	Male	86	Married	Worker	Secondary school
P2	Female	88	Married	Housewife	Primary school
P3	Female	89	Married	Housewife	Primary school
P4	Male	90	Married	Farmer	Secondary school
P5	Female	88	Married	Housewife	Primary school
P6	Female	89	Married	Housewife	Primary school
P7	Female	59	Married	Tradesman	Secondary school
P8	Female	52	Married	Housewife	Secondary school
P9	Female	53	Married	Housewife	Secondary school
P10	Male	90	Married	Driver	Secondary school
P11	Male	63	Married	Carpenter	Secondary school
P12	Female	52	Married	Housewife	Secondary school
P13	Male	50	Married	Association manager	Undergraduate
P14	Female	88	Married	Housewife	Primary school
P15	Female	89	Married	Housewife	Primary school
P16	Male	90	Married	Farmer	Secondary school
P17	Female	88	Married	Housewife	Primary school
P18	Female	88	Married	Housewife	Primary school

Table 1 shows the demographic characteristics of the 18 participants included in the study. Of the participants, 67% (12 individuals) were women and 33% (6 individuals) were men, all residing in the center of Ahlat. The relatively high number of female participants was considered significant because women are generally the primary preservers and transmitters of culinary traditions within households. Male participants, on the other hand, contributed important narratives particularly within the framework of oral history and migration memory. The average age of the participants was notably high. Twelve participants were between 86 and 90 years old, indicating that many had directly experienced the 1944 Ahiska exile during childhood or belonged to the first generation to witness its immediate consequences. This age profile enhanced the historical depth and authenticity of the findings. Regarding education level, the majority of participants were graduates of primary school or secondary school. Rather than representing a limitation, this educational profile reflects the predominantly oral transmission of Ahiska culinary knowledge, in which recipes, preparation techniques, and food-related rituals have traditionally been transferred within families practices rather than formal written documentation. Consequently, participants were considered particularly suitable for documenting the continuity of culinary heritage and migration memory.

### 3.4 Data analysis

The interview transcripts were analysed using qualitative content analysis to identify recurring meanings, concepts, and culturally significant patterns within participants’ narratives. An inductive and iterative coding procedure was adopted, allowing themes to emerge directly from the data rather than relying on predetermined categories. Throughout the analysis, constant comparison was employed to examine similarities and differences across participants’ narratives.

The analysis proceeded in four stages. First, all transcripts were read repeatedly to gain familiarity with the data and to obtain a comprehensive understanding of participants’ migration experiences, culinary memories, and food-related practices. During this stage, analytical notes and memos were prepared to identify meaningful expressions relevant to the research objectives.

Second, open coding was conducted by assigning descriptive codes to meaningful words, sentences, and passages. As the coding process progressed, similar codes were continuously compared and revised to improve conceptual consistency.

Third, conceptually related codes were grouped into broader categories and subsequently synthesized into five overarching themes: Migration, Food and Beverage, Culinary Practices, Events, and Traditions. The relationships among codes, categories, and themes were repeatedly reviewed to ensure conceptual coherence and clear distinctions between themes.

Finally, representative quotations were selected to preserve participants’ original expressions and to support the interpretation of the findings. The coding framework was refined through continuous comparison of newly coded data with previously coded segments until thematic saturation was achieved and no substantially new concepts emerged from the data.

### 3.5 Trustworthiness of the study

Several strategies were employed to enhance the trustworthiness and methodological rigor of the study. First, investigator triangulation was implemented throughout the coding process. Two researchers independently coded the interview transcripts and subsequently compared their coding decisions. Differences were discussed until consensus was reached, and the final coding framework was established collaboratively, thereby strengthening analytical consistency and reducing subjective interpretation. Second, an audit trail was maintained throughout the research process. All stages of data collection, transcription, coding, category development, and theme generation were systematically documented to ensure transparency and traceability of the analytical procedures. Third, credibility was enhanced through the use of direct participant quotations in the findings section, allowing readers to evaluate the relationship between participants’ narratives and the interpretations presented by the researchers. Finally, participant confidentiality was ensured through the use of identification codes (P1–P18), and all ethical principles concerning voluntary participation, anonymity, and informed consent were strictly observed.

## 4 Findings

### 4.1 Migration

**FIGURE 5:**
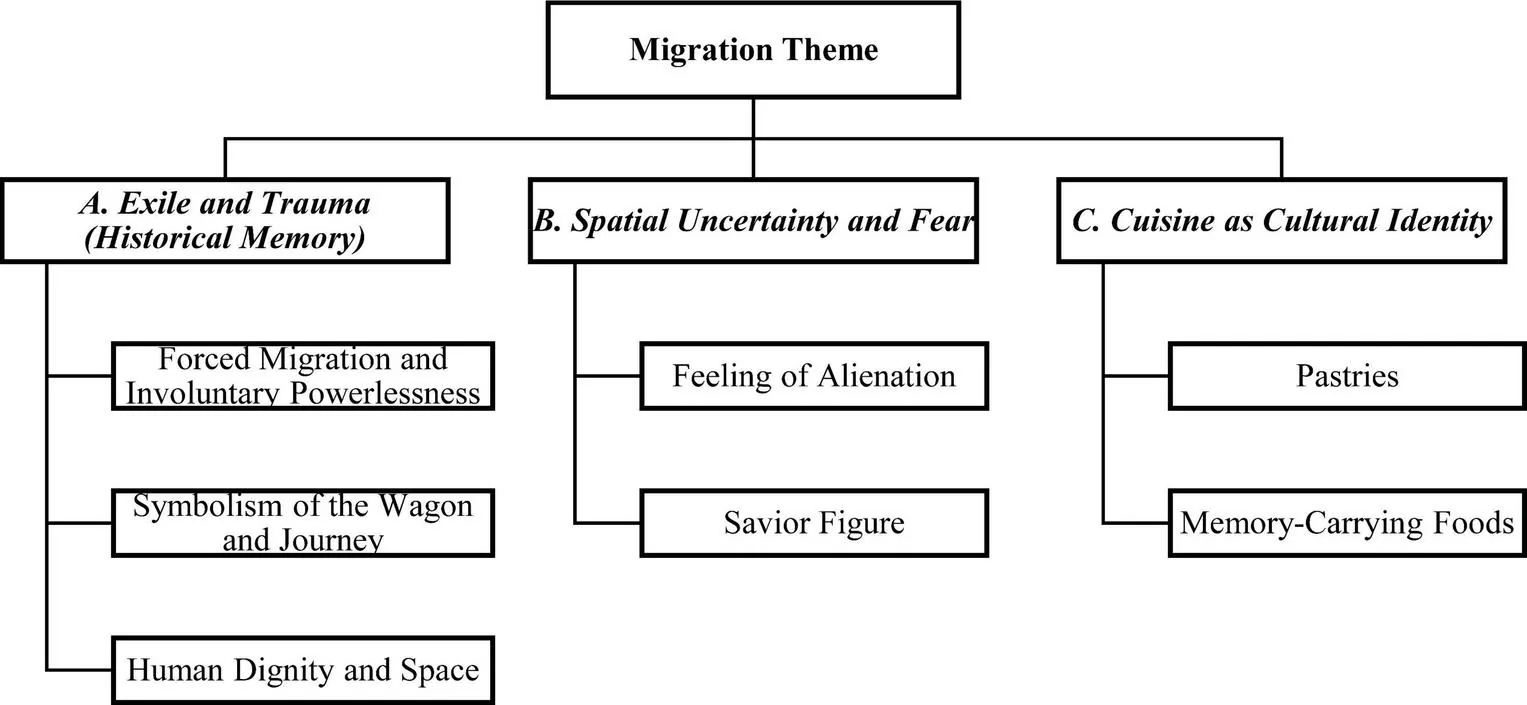
The theme of migration in the cuisine of the Ahiska exiles.

The first theme of the research, migration, aimed to create a collective memory regarding past and present challenges and difficulties by determining what the Ahiska exile and its cuisine meant to the participants. In this context, participants were asked the following fundamental questions: “In what year did you settle in this region? Why did you choose this region? What challenges or advantages did you encounter? What meaning/connotations does Ahiska cuisine hold for you?” The migration theme and its sub-dimensions are presented in Figure 5 within the framework of these fundamental questions.

When Figure 5 is examined, the sub-dimensions of the migration theme were referenced as follows: Exile and Trauma (Historical Memory) was raised by six participants (P1, P2, P3, P4, P10, P14), Spatial Uncertainty and Fear by four participants (P5, P6, P10, P16), and Cuisine as Cultural Identity by nine participants (P7, P8, P9, P11, P12, P13, P15, P17, P18). As several participants referred to more than one sub-dimension, these figures indicate the number of participants raising each theme rather than mutually exclusive proportions.

A. Exile and Trauma (Historical Memory)

Forced Migration and Involuntary Powerlessness: Almost all participants began their statements by saying, “It wasn’t our personal choice,” “I didn’t want to,” or “We have to go.” Therefore, this situation underscores a collective victimization and displacement by the state.

Symbolism of the Wagon and Journey: The most common phrases used by participants in their accounts were “closed wagons,” “train station,” and “a journey lasting 45–50 days.” These are common keywords representing the physical hardship of the exile (hunger, disease, death, etc.).

Human Dignity and Space: The expressions “They dumped them in the animal market” and “They turned the barns into homes” illustrate the inhumane treatment and struggle for survival (their efforts to adapt) faced by the exiled people.

B. Spatial Uncertainty and Fear

Feeling of Alienation: This reveals the feelings of “fear” and “insecurity” that come with being a child in a geographical area (Central Asia) that the participants are unfamiliar with.

Savior Figure: The name “Osman Yusufov,” mentioned by P4 and P16, has become ingrained in collective memory as a figure who prevented a catastrophe.

C. Cuisine as Cultural Identity

Pastries: Most participants associated Ahiska culinary culture primarily with pastry-based foods. For example, one participant explained, “Wherever we went, we always made kete and hinkal; they reminded us of home” (P11).

Memory-Carrying Foods: For many, traditional dishes are not merely a means of nourishment but also symbols of a homeland lost through exile and a cultural identity that has been preserved.

In this context, even though 82 years have passed since the exile, the trauma caused by the Ahiska exile is still vividly remembered. The interviews revealed that the participants reached Türkiye through a series of migration movements. The prominent elements stemming from the migration due to the exile include forced migration, the long train journey, the vivid memory of the hardships experienced during this journey, the fear of death, concerns about personal safety, anxiety, hunger, and the inhumane practices endured. Accordingly, the most striking consequences of the exile are the hardships experienced. Another key finding is that Ahiska cuisine is an important part of their traditional culture, and that pastry-based foods are widely used within Ahiska cuisine. Overall, the findings suggest that participants perceive Ahiska cuisine not only as a collection of traditional foods but also as a cultural resource through which memories of forced migration and collective identity have been preserved across generations.

These findings demonstrate that culinary practices function as a mechanism of cultural memory. In particular, the continued preparation of traditional dishes reveals that Ahiska Turks maintain symbolic ties to their homeland, despite currently living in various locations. In this context, the findings suggest that local cuisine enables displaced communities such as the Ahiska Turks to preserve their sense of belonging and collective identity. These findings are consistent with the existing literature on diaspora food culture.

As one participant stated, “We lost our homeland, but we never forgot our food” (P7). These findings demonstrate that memories of exile are not merely viewed as traumatic experiences belonging to the past but are actively sustained through daily culinary practices. Consequently, the preparation and consumption of food function as mechanisms that extend beyond the mere satisfaction of nutritional needs; they enable the Ahiska Turks to maintain ties to their lost homeland and to reconstruct their collective identities.

### 4.2 Food and beverage

The second theme of the research, food and beverages, aims to categorize the dishes, products, and beverages in Ahiska cuisine. In this context, participants were asked the fundamental questions: “What are the dishes made in Ahiska cuisine?, What are the products prepared in Ahiska cuisine?, What are the beverages unique to Ahiska cuisine?” This aims to identify the prominent food and beverage products in Ahiska cuisine (Table 2).

**TABLE 2:** Food and beverage products.

Foods	F	Category	Beverages	F	Key feature
Hinkal	10	Pastries / dumplings	Compote (general)	14	The most basic fruit drink
Samsa	9	Pastries / bread-sweet	Ayran	9	The most common dairy product/refreshing
Kete	8	Pastries / bread	Pear (compote)	8	Leader in fruit preference
Sinor (Sinhar)	8	Pastries / dough-based	Sherbet	5	Traditional sweet drink
Bishi	7	Pastries / fried	Tea	3	Daily consumption
Papa	7	Pomade / dish (corn or wheat flour)	Ganpot (compote)	1	Caucasian/old language influence
Haçapur and Kikil	6	Pastries / snacks	Cranberry / rosehip	1	Medicinal/Sour flavors
Lobiya and Cadi	5	Legumes (beans) and corn bread	Chacha (homemade)	1	Traditional distilled spirit

The frequencies (f) presented in the tables represent the number of participants who referred to a specific food item, culinary practice, or cultural element during the interviews. In thematic figures, percentages show the relative distribution of coded references within each main theme rather than statistical prevalence, because participants mentioned more than one concept during the interviews. Table 2 shows that six of the eight most frequently cited food items were dough-based products (Hinkal, *f* = 10; Samsa, *f* = 9; Kete, *f* = 8; Sinor, *f* = 8; Bishi, *f* = 7; Haçapur/Kikil, *f* = 6). These findings may reflect dietary preferences shaped by agricultural production patterns and the environmental conditions experienced by Ahiska communities. The food names reflect a blend of Caucasian (Khachapur, Hinkal, Lobiya) and Black Sea and Anatolian (Cadi, Erişte) culinary traditions. Papa, consumed as a staple food with high satiety alongside the main meal or at breakfast (frequency 7 times), is an important complementary food. Soup varieties (Tutmaç, Ovalama) constitute the “starter” elements of the traditional table setting. In summary, the main flavors of Ahiska culinary culture are: main fillings such as Hinkal, Pilaf, Uzbek Pilaf, Erişte, and Karnıcırık; dough-based and bread derivatives such as Kete, Bazlama, Cadi, and Bishi; and traditional snacks such as Sinor, Khachapur, Kikil, Katmer, and Makarama. Lobiya (beans) stand out as a source of legumes.

When beverages are examined, “Hoşaf” stands out as the most characteristic product. This indicates that the tradition of drying fruits and preparing for winter is very strong in the region. Other beverages include products such as “Şerbet,” “Kızılcık,” and “Kuşhipu,” which are either entirely gathered from nature or prepared at home. In addition, the word “Ganpot” is a term used for compote, articularly common around the Caucasus/Ahiska region. “Çaça” refers to a strong alcoholic drink made from grape or fruit pulp in the region. Participants’ narratives suggest that these food and beverage practices play an important role in maintaining family interaction and cultural continuity, particularly during periods of hardship. As P15 stated, “When everyone gathered around the same table, it felt like we were together again.” Therefore, these foods and beverages can be regarded as symbolic resources through which collective identity and memory are transmitted from generation to generation. In this context, the findings suggest that immigrant communities view their culinary heritage as a significant tool for preserving intangible cultural heritage. In this context, it is seen that the list of food and beverages is not a random collection of dishes, but rather reflects the spirit of a specific geography and represents the strongest social bond that keeps families together during difficult times.

### 4.3 Culinary practice

**FIGURE 6:**
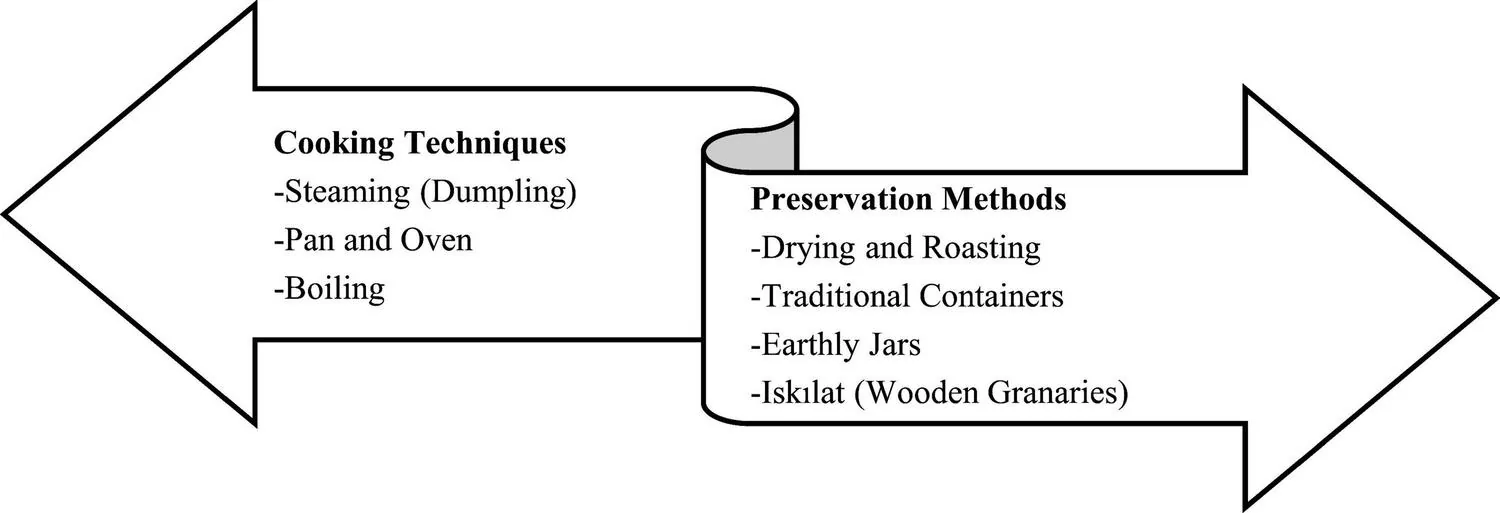
Storage methods and cooking techniques.

The third theme of the research, focusing on culinary practices, aimed to identify the cooking techniques, preservation methods, and kitchen utensils unique to Ahiska cuisine. In this context, participants were asked three fundamental questions: “Is there a cooking technique unique to Ahiska cuisine?”, “Is there a preservation technique unique to Ahiska cuisine?”, and “Is there a kitchen utensil unique to Ahiska cuisine?”. This allowed researchers to uncover the basic practices of Ahiska cuisine, initially examining cooking techniques and preservation methods (Figure 6).

When Figure 6 is examined, steaming was by far the most frequently cited cooking method, mentioned by nearly all participants. This shows how deeply rooted and beloved the “Hinkal” culture is in Ahiska cuisine. The fact that the dough is cooked in steam rather than in water helps it retain its shape. The use of griddle reflects the nomadic/highland culture, while the oven reflects the settled baking tradition. Finally, boiling is a complementary technique, especially for pasta, noodles, and dumplings. Regarding storage methods, the harsh climate and forced migration have created a need to preserve food for extended periods. This has led Ahiska people to adopt important storage techniques. Drying and roasting are fundamental to winter preparation. The use of traditional wooden containers (külek, boçka, fıçı) for storing cheese and butter adds a characteristic aroma to the food. Earthenware jars provide thermal insulation, preventing food spoilage. In addition, wooden granaries where grains and dry goods were stored represent an important part of culinary culture. Following cooking techniques and storage methods in the context of culinary practices, the purpose and importance of traditional kitchen utensils were determined (Table 3).

**TABLE 3:** Kitchen utensils.

Kitchen Utensils	Purpose of use	Importance level
Hinkal pot	Multi-layered special cauldron for steam cooking.	Critical (highest)
Copper pot / tray	High heat conductivity, suitable for mass cooking.	High
Bucket / basket	Wooden containers for storing oil, cheese and pickles.	High
Dough board / rolling pin	Preparation stage of pastries (kete, hinkal).	High
Earthly pot / jar	Slow cooking and cool storage.	Medium
Copper strainer	To remove cooked pastries without damaging them.	Medium

When Table 3 is examined, the Hinkal cauldron, as a kitchen utensil, is not just a kitchen tool, but a seal of Ahiska identity in the kitchen. This is because the participants’ narratives show that the Hinkal pot functions not merely as a cooking utensil, but as an important cultural symbol in Ahiska culinary practices. The fact that almost every participant (P1 to P18) pronounced this cauldron should be seen as the most concrete evidence of cultural continuity. The frequent reference to this utensil across participant narratives suggests its continuing symbolic significance in preserving culinary traditions. As P3 explained, “Without the hinkal pot, it does not feel like Ahiska food.” In addition, a healthy and traditional kitchen infrastructure based entirely on natural materials (copper, earth, wood), without plastic or industrial products, exists in Ahiska culinary culture. The use of traditional storage containers clearly reveals how the people strategically managed food in the face of forced migrations and harsh winters.

These findings demonstrate that traditional cooking methods not only adapt to the local ingredients and conditions of Ahlat but are also preserved sustainably. In this context, the findings suggest that cultural continuity is maintained and that culinary culture continues to evolve through interaction with the local food culture rather than through static preservation.

### 4.4 Events

The fourth theme of the research, the event theme, identifies practices that are unique to Ahiska cuisine. In this context, participants were asked three fundamental questions: “What are the special occasion dishes unique to Ahiska culinary culture?”, “Is there a specific food ritual specific to Ahiska culinary culture?”, and “Are there any food-related events or festivals specific to Ahiska culinary culture?” The aim was to uncover the unique practices of Ahiska cuisine (Table 4).

**TABLE 4:** Special practices unique to Ahiska cuisine.

Occasion	Most frequently served dishes	Symbolic meaning
Wedding	Uzbek Pilaf, Lobiya, Bride’s Hinkal	Fertility, continuation of lineage
Funeral	Flour halva, empty katmer	Patience, solidarity, remembrance
Holiday/religious festival	Bishi, kete, katmer	Sharing, announcement (scent)
Bride’s arrival	Convoy katmeri	Journey and acceptance
Send-off for soldiers	Kete	Protection and good fortune

Table 4 reveals that Ahiska cuisine is a profound tool of social solidarity and ritual, accompanying every stage of life from birth to death. Elements such as “Bride’s Hinkal” and “Funeral Halva” are reflections of collective memory in the kitchen. Therefore, in Ahiska culture, dishes are sharply distinguished according to the nature of the ritual (joy or mourning). For example, Uzbek Pilaf and Hinkal are the main dishes of weddings. P14 stated, “No wedding was complete without pilaf and hinkal.” Löbiya holds an important place as an accompaniment to pilaf in traditional wedding menus (K14, K15, K16). The tradition of distributing katmer (a type of flatbread) in the bridal procession is a lively ritual celebrating the journey and arrival home. Leaving money in the sherbet glass during engagements and betrothals is both a celebration and a symbolic act of support for the young couple about to get married. The most common mourning dish mentioned by all participants is halva. The practice of not cooking for 3 days in the house of mourning, and neighbors bringing food (especially katmer) (K2, K10, K17), reflects the strong sense of neighborliness and sharing in Ahiska culture. P2 explained, “Neighbors brought food because nobody cooked in the mourning house.” The baking and distribution of bishi (a type of flatbread) during holidays and religious festivals, to fill the house with aroma, is a way of sharing faith and tradition through a sensory element. Again, during holidays, rather than a specific rule, the principle is “to make the best of what’s available at home” (K12). Furthermore, the kitchen is a space for divination and socialization among Ahiska Turks. For example, the gender prediction based on who gets the metal coin placed in the dough transforms the meal into a game and a means of socialization. The labor-intensive preparation of hinkal, which brings neighbors together, also suggests to strengthen social bonds. The emphasis on tea and the samovar after dinner highlights the importance of the kitchen as a place for conversation.

### 4.5 Traditional

The final theme of the research, traditionalism, focuses on identifying aspects of Ahiska cuisine that were frequently prepared in the past, including meals and serving practices. In this context, participants were asked three fundamental questions: “What dishes were made in Ahiska culinary culture but are no longer made?”, “What dishes are consumed according to the meals specific to Ahiska cuisine?”, and “Is there a traditional food and beverage service specific to Ahiska culinary culture?” The aim is to reveal the prominent traditional aspects of Ahiska cuisine.

Meals: In Ahiska cuisine, meals are based on animal-based breakfasts and balanced carbohydrate and protein dinners. Breakfasts focus on dairy products and “sarı yağ” (clarified butter). Chechil cheese, clotted cream, and mihlama (a flourless version made only with cheese and butter), and Chuma (curd cheese melted in butter) are energy-boosting dishes unique to this cuisine. Lunch is generally lighter or more practical. Dinner can be considered the richest and heaviest meal of the day. “Hinkal, Löbiya, Pilav, and Papa” are staple dishes.

Forgotten dishes: The most striking finding in the analysis is “Çinçar (Nettle)” soup. The vast majority of participants (K5, K8, K11, K13, K15, K16, K17, K18) listed this soup among the dishes that are no longer made or are becoming very rare. Similarly, cooked and pumpkin soups are also on the verge of disappearing. Stuffed flatbread (kete) and “Lazut,” made from pounded corn, are less commonly made today due to the laborious preparation process.

Table etiquette and serving rituals: The Ahiska table is considered not just a place to eat, but a space of respect and generosity. Instead of Western-style “sequential serving,” the Ahiska tradition emphasizes “putting everything on the table at once.” Desserts, sweets (ganfet), main course, and pastries are all on the table simultaneously. This symbolizes the host’s generosity and the abundance of their table. The most important detail in serving hinkal is the garlic-vinegar sauce prepared with the boiling water. Dipping the hinkal in this sauce before eating is a technical ritual that ensures the integrity of the flavors. The fact that no one begins eating before the head of the household sits down at the table demonstrates a deep-rooted discipline and family bond. As P8 stated, “We always waited for the eldest person before starting the meal.” This situation demonstrates the importance given to family hierarchy and respect during communal meals. Furthermore, the serving on copper trays or wooden low tables symbolizes naturalness and tradition.

## 5 Discussion

This study, using Ahlat as a case study, examined how the culinary traditions of Ahiska Turks have been preserved, transformed, and transmitted following the 1944 forced exile. Rather than focusing solely on traditional dishes, the study explored the relationships among migration, culinary heritage, collective memory, and cultural identity.

The findings suggest that Ahiska cuisine has functioned as a “portable homeland” through which cultural identity and collective memory have been preserved despite repeated displacement. This finding is consistent with Diaz and Ore (39), who argue that cuisine enables displaced communities to symbolically reconstruct their homeland and maintain a sense of belonging after forced migration. Similarly, studies on diaspora foodways emphasize that food serves not only as a means of nourishment but also as a medium through which cultural continuity is sustained across generations (6, 7, 40–42).

The demographic structure of the research indicates that women play a key role as carriers of culinary knowledge and cultural memory. Participants’ narratives suggest that women have been primarily responsible for transferring recipes, cooking techniques, and food-related rituals within the family, while male participants contributed valuable narratives concerning migration history and oral memory. These findings are consistent with previous studies emphasizing the important role of women in safeguarding culinary heritage and intangible cultural heritage (43).

Traumatic images such as “train wagons,” “animal markets” and “hunger” which appeared in participants’ narratives, suggest that migration memories continue to shape contemporary culinary practices. Rather than representing only historical events, these memories appear to have been integrated into everyday food practices through traditional dishes such as Hinkal, Samsa, Bishi, and Kete. Similar observations have been reported in food anthropology studies, which argue that cuisine functions as a medium through which displaced communities reconstruct identity and preserve collective memory (44, 45).

In particular, participants frequently described Hinkal as one of the most recognizable symbols of Ahiska culinary identity and cultural continuity. In terms of cooking techniques, participants’ narratives indicate that steaming represents one of the distinctive preparation methods of Ahiska cuisine. Likewise, traditional food preservation techniques and storage methods reflect adaptation to harsh climatic conditions as well as long-term migration experiences (46, 47).

Socially, the findings indicate that the kitchen continues to function as an important social space where community solidarity is reinforced during life-cycle events such as births, weddings, and funerals. Likewise, participants associated the tradition of serving all dishes simultaneously with values such as sharing, hospitality, and social cohesion. These findings support UNESCO’s perspective that culinary heritage encompasses not only recipes but also the knowledge, skills, rituals, and social practices transmitted across generations (48, 49).

Another important contribution of the study concerns the interaction between Ahiska cuisine and the local culinary culture of Ahlat. Participants’ narratives indicate that Ahiska culinary traditions have largely been preserved through gradual adaptation to the local food environment. Rather than replacing local culinary traditions, the findings suggest a process of cultural continuity and culinary adaptation in which migrant and local food cultures coexist and mutually influence one another (50). This finding contributes to the literature on diaspora cuisine by demonstrating that migrant cuisines remain dynamic cultural systems rather than static collections of traditional recipes (51).

Despite this continuity, participants also expressed concern regarding the gradual disappearance of several traditional dishes, ingredients, and cooking practices. The findings suggest that modernization, changing lifestyles, and decreasing intergenerational transmission may threaten the long-term sustainability of Ahiska culinary heritage.

## 6 Conclusion

This study contributes to the international literature on culinary heritage, diaspora foodways, food anthropology, and cultural memory by providing empirical evidence from an underexplored migrant community. The findings demonstrate that cuisine represents not only a collection of traditional recipes but also a dynamic cultural system through which identity, memory, and belonging are continuously reconstructed following forced migration. Our research findings regarding Ahiska cuisine based on the example of Ahlat reveal that it functions, in a sense, as a “portable homeland,” serving to sustain a sense of belonging and reflecting an effort to symbolically reconstruct the homeland following forced migration.

The research has revealed that women are the primary bearers of culinary knowledge and the preservers of intangible cultural heritage. Traumatic migration memories such as “train wagons” and “hunger” found in the participants’ narratives have been integrated into contemporary culinary practices through traditional dishes like Hinkal and Kete. Hinkal, in particular, is identified as the strongest symbol of Ahiska identity, and its steaming technique stands out as a distinctive element of the culinary culture.

The interaction between the cuisines of Ahiska and Ahlat should be viewed not as a cultural displacement, but as a process of cultural adaptation and hybridization. The findings demonstrate that immigrant cuisine is not a static collection of recipes; rather, it is a dynamic system that is constantly reconstructed through interaction with the local culture.

From a practical perspective, the findings indicate that documenting traditional recipes, cooking techniques, and food-related rituals through oral history projects, digital gastronomy archives, and community-based cultural initiatives may contribute to safeguarding this intangible cultural heritage. Furthermore, selected elements of Ahiska cuisine may be incorporated into local gastronomy tourism and cultural heritage programmes while preserving their cultural authenticity.

Several limitations should be considered when interpreting the study. First, the research was conducted only in the Ahlat district of Bitlis Province; therefore, the findings cannot be generalized to all Ahiska communities. Second, the study involved a purposive sample of eighteen participants, most of whom belonged to the older generation. Although this participant profile was particularly valuable for documenting migration memories and traditional culinary knowledge, younger generations may attribute different meanings to Ahiska cuisine. Finally, the findings are based on retrospective narratives, which may be influenced by memory and personal interpretation.

Future research may compare Ahiska communities living in different regions of Türkiye and other countries to examine similarities and differences in culinary adaptation and cultural continuity. Comparative studies focusing on diaspora foodways, intergenerational transmission, gastronomic tourism, geographical indications, and consumer perceptions would further strengthen the international literature on migrant culinary heritage. The cuisines of societies play a vital role as mechanisms for preserving identity and fostering social solidarity.

## Data Availability

The original contributions presented in this study are included in this article, further inquiries can be directed to the corresponding author.

## References

[1] Tamer Y. *Social Cohesion in Forced Migration: an Attempt to Operationalize Social Cohesion for Syrians Under Temporary Protection in TURKEY.* Master’s thesis, Turkey: TED University (2021).

[2] Hunkler C Scharrer T Suerbaum M Yanasmayan Z. Spatial and social im/mobility in forced migration: revisiting class. *J Ethnic Migrat Stud.* (2022) 48:4829–46. 10.1080/1369183X.2022.2123431

[3] Law L. Home cooking: filipino women and geographies of the senses in Hong Kong. *Ecumene.* (2001) 8:264–83. 10.1177/096746080100800302

[4] Ray K. *The Migrants Table: Meals and Memories in.* Philadelphia, PA: Temple University Press (2004).

[5] Oğan Y Uzman M. Reflections of Turkey-Greece population exchange on Manisa Alaşehir culinary. *Intern J Gastron Food Sci.* (2025) 37:101309. 10.1016/j.ijgfs.2025.101309

[6] Avieli N. What is ‘Local Food?’ dynamic culinary heritage in the world heritage site of hoi an, Vietnam. *J Heritage Tour.* (2013) 8:120–32. 10.1080/1743873X.2013.767812

[7] Hedi Nairi A. Food and foodways of the maghrebian diaspora in turkey: identity, belonging, and integration. *Moment Dergi.* (2023) 10:176–99. 10.17572/mj2023.1.176199

[8] Avşar Z Tunçalp Z. *Sürgünde 50. Yıl Ahiska Türkleri.* Ankara: TBMM Yayını (1994).

[9] Enders Wimbush S Wixman R. The meskhetian turks: a new voice in Soviet Central Asia. *Can Slavonic Papers.* (2015) 17:320–40. 10.1080/00085006.1975.11091412

[10] Kalkan M. Türk-Moğol Kavimleri Arasında Tatarlar ve Menşeî Meselesi. *Türk Kültürü Dergisi.* (1996) 393:11–8.

[11] Zeyrek Y. *Ahiska Bölgesi ve Ahiska Türkleri.* Ankara: Pozitif Matbaacılık (2001).

[12] Veyseloğlu AP. *Ahiska Türkleri’nin Dramı.* Ankara: Ocak Yayınları (1999). p. 1–224.

[13] Yalçınkaya A. *Kafkasya’da Siyasi Gelişmeler Etnik Düğümden Küresel Kördüğüme.* Ankara: Lalezar Yayınları (2006).

[14] Yüzbey İ. Ahiskalı Türkler ve kültürleri. *Turkish Stud.* (2008) 3:679–706. 10.7827/TurkishStudies.525

[15] Taşdemir T. *Türkiye’nin Kafkasya Politikasında Ahiska ve Sürgün Halk Ahiskalılar.* İstanbul: IQ Kültür-Sanat Yayıncılık Araştırma (2005).

[16] Kırzıoğlu MF. *1855 Kars Zaferi.* İstanbul: Kars Tanıtma Derneği Yayınları (1955).

[17] Baddeley JF. *Rusların Kafkasya’yı İstilası ve ϸeyh ϸamil.* İstanbul: Çev.: Sedat Özden, Kayıhan Yayınevi (1989).

[18] Kahvecioğlu Sarı H. Honaz’da Yaşayan Ahiska Türklerinin Düğün Törenleri Üzerine Bir Araştırma. *J Soc Sci.* (2017) 13:205–15. 10.16990/SOBIDER.3673

[19] Onyango E Owuor PM Otoadese D Odhiambo SA Ajibade TB Onyango JA et al. Beyond conventional metrics: a scoping review of cultural food security definitions and indicators for migrant populations. *Front Nutr.* (2026) 13:1841239. 10.3389/fnut.2026.1841239 42540478 PMC13426276

[20] Kyzy CZ Sormaz Ü. Ahiska Türkleri’nin Yemek Kültürü Üzerine Bir Araştırma. *J Turkic Civil Stud.* (2025) 6:76–95.

[21] Seçim Y Kaya M. Türkiye’de yaşayan Ahiska Türklerinin yemek kültürü. *Türk Turizm Araştırmaları Dergisi.* (2019) 3:1631–47. 10.26677/TR1010.2019.262

[22] Badalov C. Bütün Yönleriyle Ahiska Türkleri. Gasip-S Yayinlari: Çimkent (2004).

[23] Bayraktar R. *Ahiska-Çıldır Beylerbeyliği.* İstanbul: Birleşik Yayıncılık (2000).

[24] ULUSAM. *Ahiska location*. (2021). Available online at: https://www.ulusam.org.tr/?s=Ah%C4%B1ska

[25] Kahraman SÖ İbrahimov A. Kafkaslar’dan sürgün bir toplumun bitmeyen göçü: Çanakkale’de Ahıska Türkleri. *Ege Coğrafya Dergisi.* (2013) 22:77–90.

[26] Serverov Y. *Ben Ahiskalı Bir Türküm.* Moskova: Soyuz Yayınevi (1990).

[27] Gürbüz D Kul M. Bir Sürgünün Anatomisi: Ahiska Türklerinden Bekir Mamoyev ile Sözlü Tarih Çalışması. *Akademik Bakış Uluslararası Hakemli Sosyal Bilimler Dergisi.* (2016) 55:132–45. https://izlik.org/JA76DD88EL

[28] Ediev D. *Demografçeskie Poteri Deportirovannıh Narodov SSSR.* Stavropol: AGRUS (2003).

[29] Çelikpala M. Başarısız devlet-demokratik model ülke sarmalında Gürcistan’ın 20 yılı. *Orta Asya Kafkasya Araştırmaları.* (2012) 14:1–35.

[30] Bugay NF. *L.Beriya-İ.Stalinu: “Soglasno Vaşemu Ukazaniyu”.* Moskva: Airo-XX (1995).

[31] Directorate of Migration Management Available online at: https://www.goc.gov.tr/ (accessed Februaruy 08, 2026) (2026).

[32] Elmastaş N. Ahlat Yöresi’nin Turizm Potansiyeli. *Marmara Coğrafya Dergisi.* (2001) 3:153–82.

[33] Karasar N. *Bilimsel Araştırma Yöntemi. (12. Baskı).* Ankara: Nobel Yayıncılık (2003).

[34] Singleton RA Straits BC. *Approaches to Social Research.* New York, NY: Oxford University Press (2005).

[35] Yıldırım A Şimşek H. *Sosyal Bilimlerde Nitel Araştırma Yöntemleri.* Ankara: Seçkin Yayıncılık (2005).

[36] Baltacı A. The qualitative research process: how to perform a qualitative research? *Ahi Evran Üniv Sosyal Bilimler Enstitüsü Dergisi.* (2019) 5:368–88. 10.31592/aeusbed.598299

[37] Merriam SB Grenier RS. *Qualitative Research in Practice: Examples for Discussion and Analysis.* San Francisco, CA: Jossey-Bass Publishers (2019).

[38] Guest G Bunce A Johnson L. How many interviews are enough? An experiment with data saturation and variability. *Field Metho.* (2006). 18:59–82. 10.1177/1525822X05279903

[39] Diaz CJ Ore PD. Landscapes of appropriation and assimilation: the impact of immigrant-origin populations on US cuisine. *J Ethnic Migration Stud.* (2022) 48:1152–76. 10.1080/1369183X.2020.1811653

[40] Long LM. Learning to listen to the food voice: recipes as expressions of identity and carriers of memory. *Food Culture Soc.* (2004) 7:118–22. 10.2752/155280104786578067

[41] Sutton DE. Food and the senses. *Ann Rev Anthropol.* (2010) 39:209–23. 10.1146/annurev.anthro.012809.104957

[42] Mannur A. Ear, dwell, orient: food networks and Asian/American cooking communities. *Cultural Stud.* (2012) 27:585–610. 10.1080/09502386.2012.725060

[43] Bessière J. Local development and heritage: traditional food and cuisine as tourist attractions in rural areas. *Sociol Ruralis.* (1998) 38:21–34. 10.1111/1467-9523.00061

[44] Counihan C Van Esterik P. *Food and Culture.* 3rd ed. New York, NY: Routledge (2013).

[45] Holtzman J. *Uncertain Tastes: Memory, Ambivalence, and the Politics of Eating in Samburu, Northern Kenya.* Oakland, CA: Univ of California Press (2009).

[46] Ranta R Ichijo A. *Food, National Identity and Nationalism: From Everyday to Global Politics.* Cham: Palgrave Macmillan (2022).

[47] Crenn C Marchiset S Téchoueyres I. Food solidarities for and with migrants: mobilizations under pressure in public spaces in the Bordeaux area. *Food Culture Soc.* (2025) 28:267–86. 10.1080/15528014.2024.2350141

[48] Lingham-Tsiaparas S Maye D Manning L. Cultural value or food culture: critiquing the importance of vocabulary, context and meaning in food studies. *Gastronomy.* (2026) 4:12. 10.3390/gastronomy4020012

[49] Sammells CA. Haute traditional cuisines: How UNESCO’s list of intangible heritage links the cosmopolitan to the local. In: Brulotte R. Di Giovine MA., editors. *Edible Identities: Food as Cultural Heritage*. Milton Park: Routledge (2016). 141–58. 10.4324/9781315578781

[50] Graham RS Hodgetts D Stolte OEE. Dual-heritage households: Food, culture, and re-membering in Hamilton. *Intern Rev Soc Res.* (2016) 6:4–14. 10.1515/irsr-2016-0002

[51] Haboucha R. Reimagined Community in London: The Transmission of Food as Heritage in the Afghan Diaspora. In: Matta R de Suremain C-E Crenn C editors. *Food Identities at Home and on the Move.* Milton Park: Routledge (2020). p. 34–48. 10.4324/9781003085430

